# Supplementary material for: Phase I Dose Escalation Study with Expansion Cohort of the Addition of Nab-Paclitaxel to Capecitabine and Oxaliplatin (CapOx) as First-Line Treatment of Metastatic Esophagogastric Adenocarcinoma (ACTION Study)
Source: Cancers (Basel). 2019 Jun 14;11(6):827. doi: 10.3390/cancers11060827 (PMC6627561; doi:10.3390/cancers11060827)
Supplement: Supplementary file 1 [file cancers-11-00827-s001.pdf]

Supplementary Materials: Phase I Dose Escalation Study with Expansion Cohort of the Addition of Nab-Paclitaxel to Capecitabine and Oxaliplatin (CapOx) as First-Line Treatment of Metastatic Esophagogastric Adenocarcinoma (ACTION Study)

Sandor Schokker, Stephanie O. van der Woude, Jessy Joy van Kleef, Daan J. van Zoen, Martijn G. H. van Oijen, Banafsche Mearadji, Ludo F. M. Beenen, Charlotte I. Stroes, Cynthia Waasdorp, R. Aarti Jibodh Aafke Creemers, Sybren L. Meijer, Gerrit K. J. Hooijer, Cornelis J. A. Punt, Maarten F. Bijlsma and Hanneke W. M. van Laarhoven

Table S1. Previous treatment and biopsies of metastatic sites.

Patient	Primary Tumor	Metastatic Sites	Previous Treatment	Surgery	Biopsy Site	CT/US Guided
1	Esophagus	Liver	CROSS	No	Liver	US
2	Esophagus	Liver, lymph nodes	None	No	Liver	US
3	Gastric	Lymph nodes, lung, local recurrence	(Neo)Adjuvant ECC	Distal stomach resection	Lymph node	US
4	GEJ	Lymph nodes	None	No	Lymph node	CT
5	Esophagus	Liver, lung	None	No	Liver	US
6	Gastric	Liver, lymph nodes	None	No		
7	GEJ	Liver, lymph nodes, adrenal	None	No	Lymph node	Excision lymph node
8	Esophagus	Liver	None	No	Liver	US
9	Esophagus	Liver, lymph nodes	CROSS	No		
10	GEJ	Lymph nodes	None	No		
11	GEJ	Lymph nodes, testes	dCRT	Esophagus-cardia resection	Testes	Orchidectomy
12	GEJ	Bone	CROSS	No	Bone	CT
13	GEJ	Lymph nodes	None	No		
14	GEJ	Lymph nodes, peritoneum	None	No		
15	GEJ	Lymph nodes, lung, local recurrence	CROSS	Esophagus-cardia resection		
16	GEJ	Liver	CROSS	Esophagus resection	Liver	US
17	Esophagus	Liver, lymph nodes, lung	CROSS	Esophagus resection	Liver	US
18	Esophagus	Lymph nodes	None	No		
19	Esophagus	Peritoneum, rectus abdominus	CROSS	No		
20	GEJ	Lymph nodes, lung, bone	None	No		
21	Gastric	Peritoneum, lymph nodes	None	No	Liver	US
22	GEJ	Lymph nodes	None	No		
23	GEJ	Liver	None	No		
24	GEJ	Lymph nodes, lung, bone, spleen, adrenal	CROSS	Esophagus resection	Lymph node	US
25	Esophagus	Liver, lymph nodes	None	No		
26	Gastric	Liver	None	No	Liver	US

GEJ: gastro-esophageal junction, CROSS: neoadjuvant chemoradiation with carboplatin/paclitaxel and 23 × 1.8 Gy radiotherapy, ECC: epirubicin, cisplatin, capecitabine, dCRT: definitive chemoradiation with carboplatin/paclitaxel and 28 × 1.8 Gy radiotherapy. This patient first had surgery without neoadjuvant treatment and subsequently dCRT for local recurrence before diagnosis of metastatic disease.

Table S2. Subsequent lines of palliative treatment.

Treatment after Action	*n*
Regorafenib/paclitaxel (REPEAT trial, ongoing, ClinicalTrials.gov Identifier NCT02406170)	6
Irinotecan	3
Ramucirumab/paclitaxel	2
Pembrolizumab (Keynote-181, ClinicalTrials.gov identifier NCT02564263)	1

Table S3. Responses to health-related quality of life questionnaires.

Condition	Screening	Before Cycle 2	Before Cycle 3 *	Before Cycle 4	Before Cycle 7	Before Cycle 10	Before Cycle 13	Before Cycle 16	Before Reintroduction	Before Cycle 2 Reintroduction
Number of returned questionnaires/ active patients in action trial (%)	24/26 (92%)	22/26 (85%)	13/25 (52%)	21/25 (84%)	19/21 (91%)	14/17 (82%)	8/10 (80%)	5/8 (63%)	4/5 (80%)	4/4 (100%)
Number of patients on CapOx-Nab-Paclitaxel	0	22	13	21	18	0	0	0	0	4
Number of patients on capecitabine monotherapy	0	0	0	0	1	14	8	5	5	0

* Before cycle 3 was an optional timepoint.

Table S4. Baseline characteristics & survival biopsy cohort.

Baseline Characteristics	Entire Cohort (*n* = 26)	Biopsy Cohort (*n* = 14)
Median age at start study (range)	63 (45–75)	63 (51–72)
Sex		
Male	23 (89%)	13 (93%)
Female	3 (11%)	1 (7%)
ECOG performance status		
0	20 (77%)	9 (64%)
1	6 (23%)	5 (36%)
Median PFS (months)	8.0	8.0
Median OS (months)	12.8	9.7

Table S5. Dose escalation scheme.

Dose Level	Nab-Paclitaxel	Capecitabine	Oxaliplatin	Minimum Number of Patients
	Day 1 and 8	14 days	Day 1 and 8	
−1	40 mg/m^2	1000 mg/m^2	65 mg/m^2	-
1 (starting)	60 mg/m^2	1000 mg/m^2	65 mg/m^2	3
2	80 mg/ m^2	1000 mg/m^2	65 mg/m^2	3
3	100 mg/ m^2	1000 mg/m^2	65 mg/m^2	3
4	120 mg/ m^2	1000 mg/m^2	65 mg/m^2	3

Table S6. Escalation decision rules.

Number of Patients with DLT at a Given Dose Level	Escalation Decision Rule
0 out of 3	Enter 3 patients at the next dose level.
1 out of 3	Enter at least 3 more patients at this dose level.<br>If 0 of these additional 3 patients experience DLT, proceed to the next dose level.<br>If 1 or more of this group suffer DLT, then dose escalation is stopped, and this dose is declared the maximally administered dose. Three additional patients will be entered at the next lowest dose level if only 3 patients were treated previously at that dose.
≥2	Dose escalation will be stopped. This dose level will be declared the maximally administered dose (highest dose administered). Three (3) additional patients will be entered at the next lowest dose level if only 3 patients were treated previously at that dose.
≤1 out of 6 at highest dose level below the maximally administered dose	This is generally the recommended phase 2 dose. At least 6 patients must be entered at the recommended phase 2 dose.

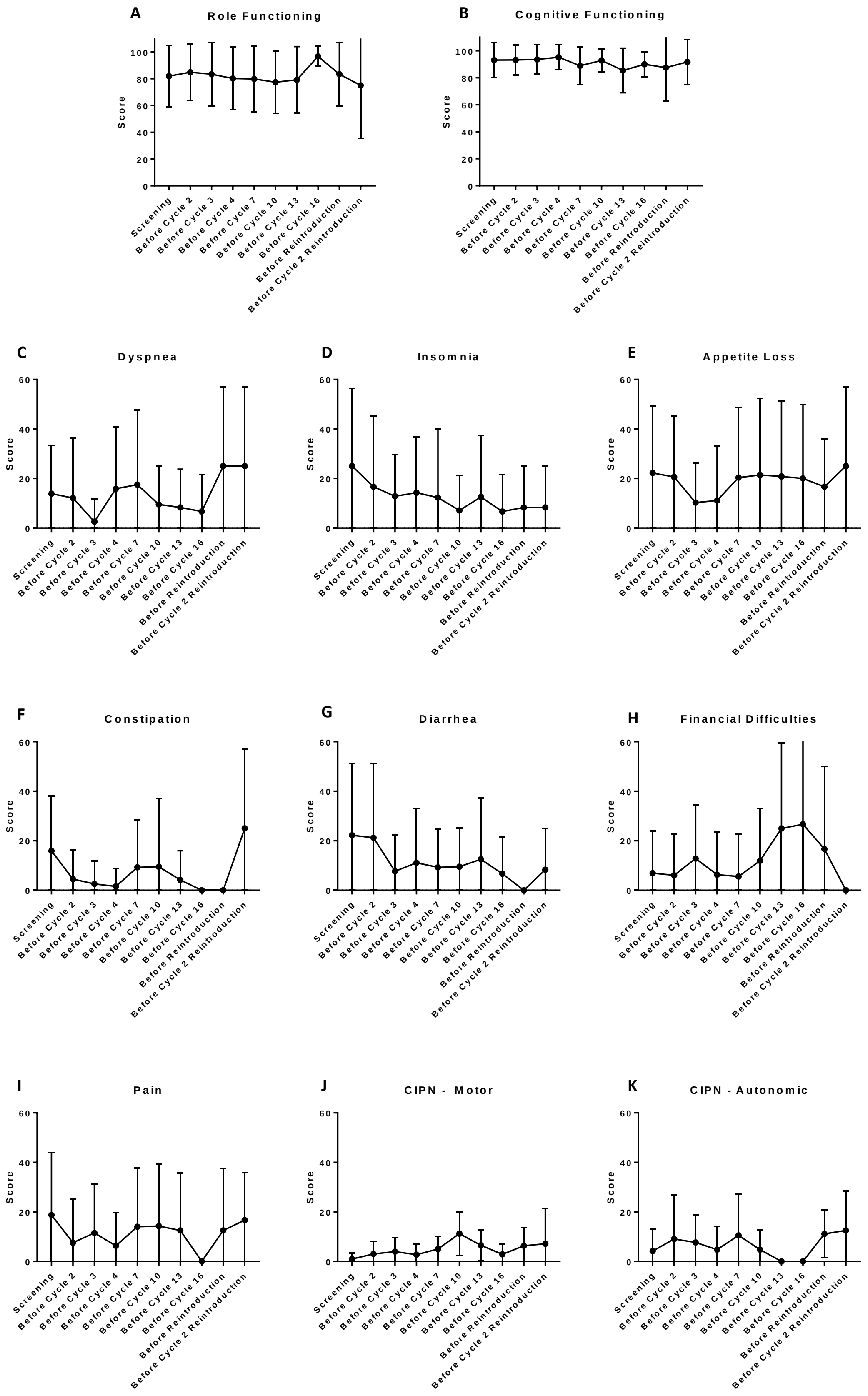

Figure S1. Health-related quality of life and patient reported neurotoxicity. Scales and single items range from 0 to 100 with high function scores denoting high level of functioning and high symptom scores denoting a high level of symptoms. CIPN: Chemotherapy Induced Peripheral Neuropathy.

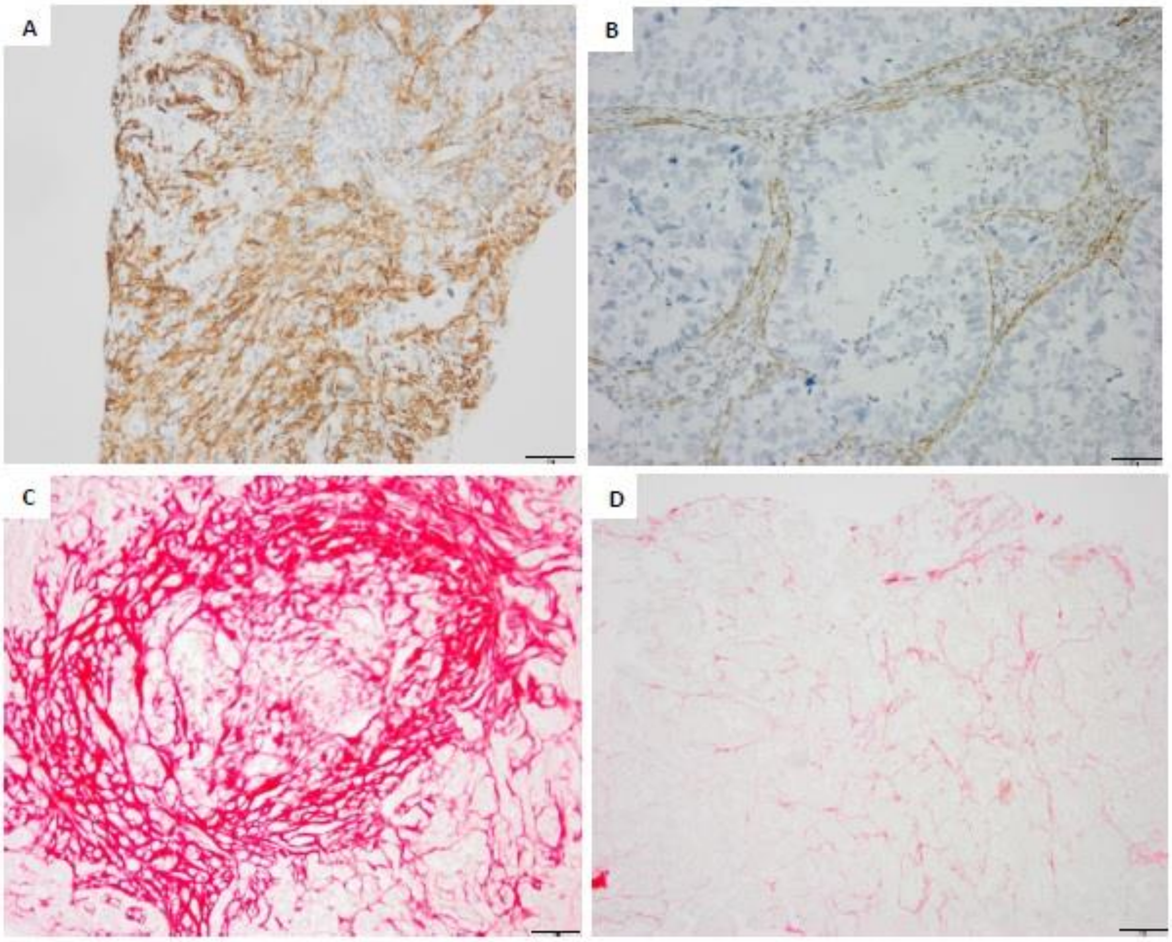

Figure S2. Representative immunohistochemistry images of αSMA and picrosirius red staining in biopsies of metastases. (**A**) αSMA high (20.7%), (**B**) αSMA low (2.0%), (**C**) picrosirius red high (42.9%), (**D**) picrosirius red low (5.3%). All images are magnified 4x and scale bar denotes 1 μm.

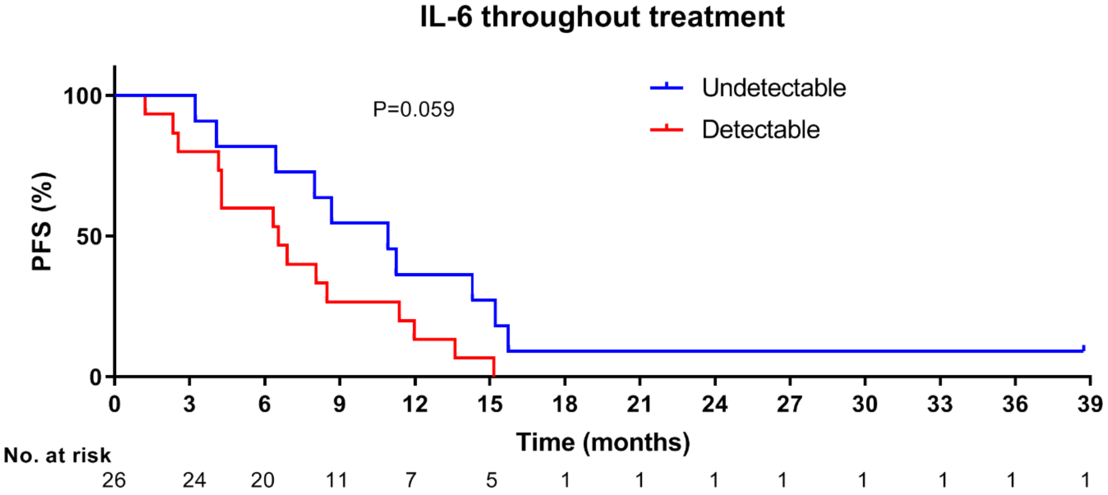

Figure S3. PFS detectable vs undetectable serum IL-6 throughout treatment. Kaplan Meier curve of progression free survival of patients with undetectable serum IL-6 throughout treatment compared with patients with one or more detectable serum IL-6 concentrations throughout treatment.

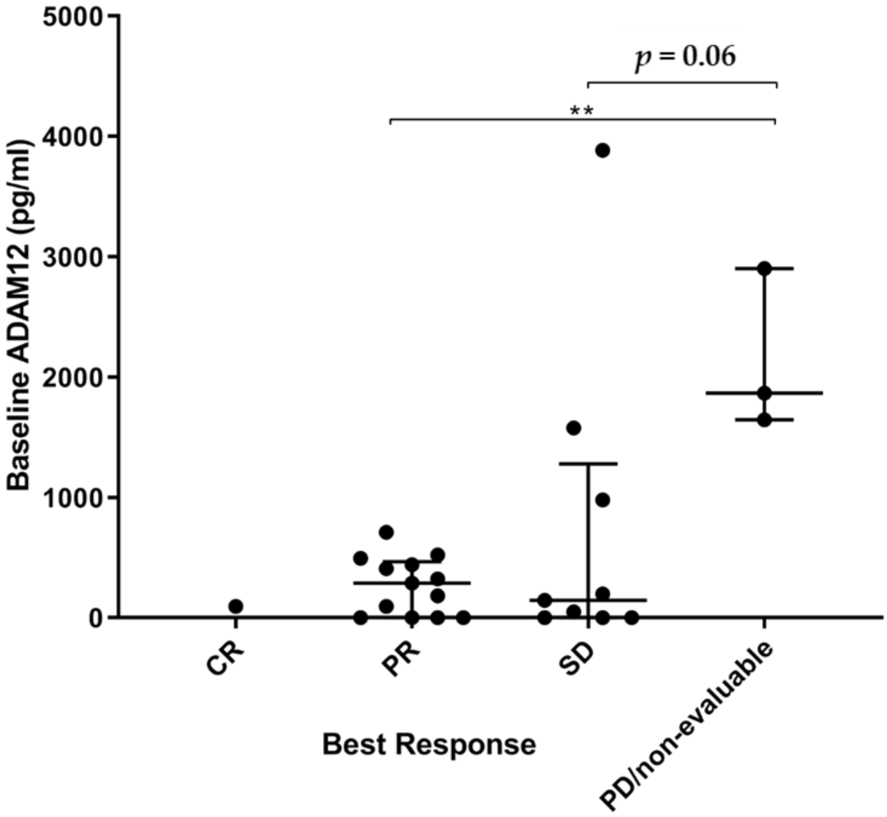

Figure S4. Best Response and baseline ADAM12. Baseline ADAM12 per best response category (with median and interquartile range). ** $p < 0.01$, CR: complete response, PR: partial response, SD: stable disease, PD: progressive disease.

A

B

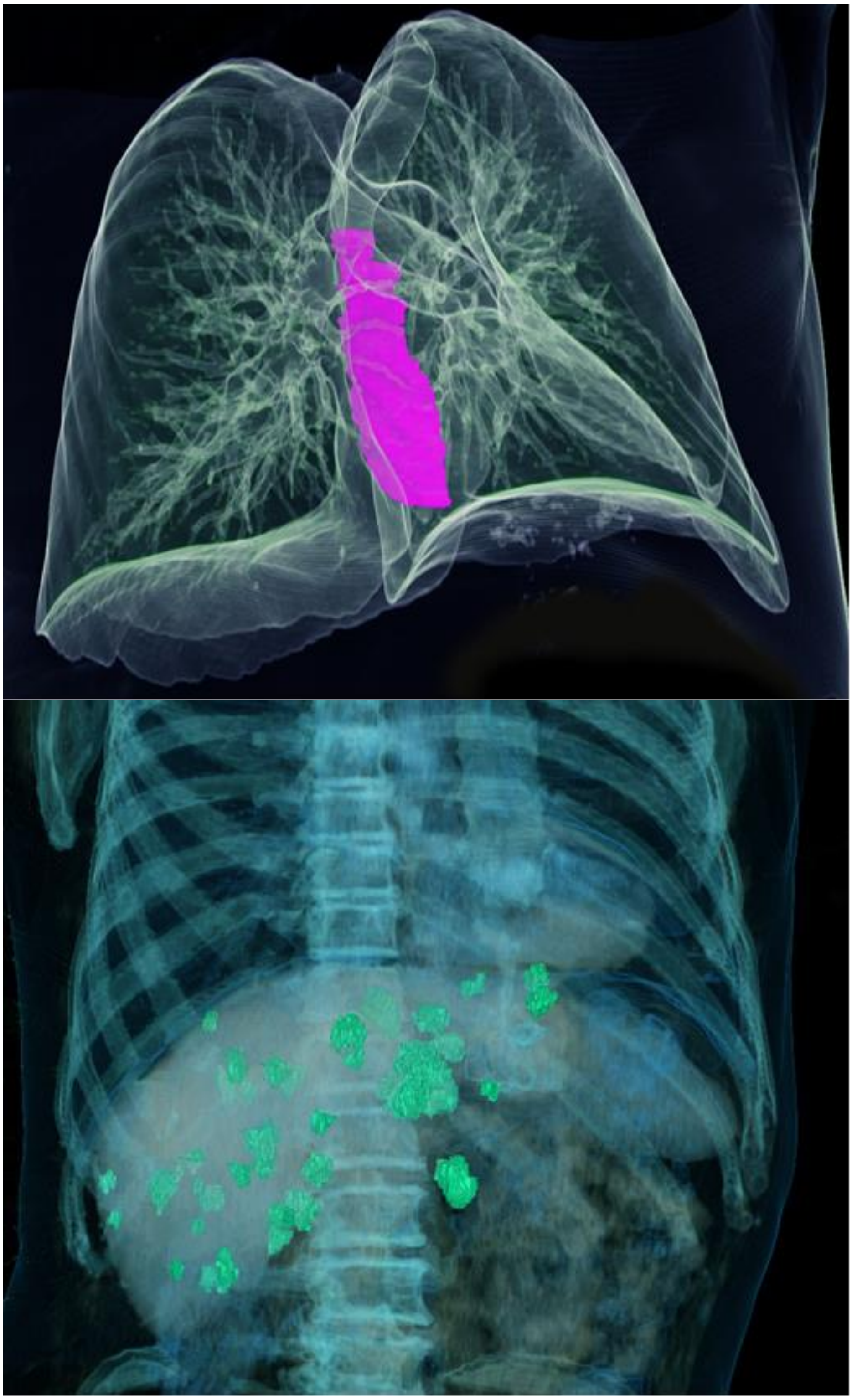

Figure S5. Representative images for primary tumor and metastases segmentation. Representative images of (**A**) primary tumor segmentation, (**B**) liver and abdominal lymph node metastases.

© 2019 by the authors. Licensee MDPI, Basel, Switzerland. This article is an open access article distributed under the terms and conditions of the Creative Commons Attribution (CC BY) license (http://creativecommons.org/licenses/by/4.0/).
